# Ketogal: A Derivative Ketorolac Molecule with Minor Ulcerogenic and Renal Toxicity

**DOI:** 10.3389/fphar.2017.00757

**Published:** 2017-11-06

**Authors:** Roberto Russo, Carmen De Caro, Bice Avallone, Salvatore Magliocca, Maria Nieddu, Gianpiero Boatto, Roberta Troiano, Rosario Cuomo, Carla Cirillo, Carmen Avagliano, Claudia Cristiano, Giovanna La Rana, Giovanni Sarnelli, Antonio Calignano, Maria G. Rimoli

**Affiliations:** ^1^Department of Pharmacy, University of Naples Federico II, Naples, Italy; ^2^Science of Health Department, School of Medicine, Magna Graecia University, Catanzaro, Italy; ^3^Department of Biology, University of Naples Federico II, Naples, Italy; ^4^Department of Chemistry and Pharmacy, University of Sassari, Sassari, Italy; ^5^Department of Clinical Medicine and Surgery, University of Naples Federico II, Naples, Italy; ^6^Laboratory for Enteric Neuroscience, KU Leuven, Leuven, Belgium

**Keywords:** ketorolac, ketogal, analgesia, gastrointestinal toxicity, histological evaluation, PAS staining

## Abstract

Ketorolac is a powerful non-steroidal anti-inflammatory drug (NSAID), with a great analgesic activity, present on the Italian market since 1991. Despite the excellent therapeutic activity, the chronic use of ketorolac has long been limited owing to the high incidence of gastrointestinal and kidney side events. In our previous study, we demonstrated that ketorolac–galactose conjugate (ketogal), synthesized and tested in a single-dose study, was able to reduce ulcerogenicity, while preserving the high pharmacological efficacy of its parent drug. In this paper, in order to verify the suitability of this compound, for repeated administration, *ex vivo* experiments on naïve mice were performed. Mice were treated for 5 or 7 days with the highest doses of two drugs (ketorolac 10 mg/kg and ketogal 16.3 mg/kg), and the expression of both gastric COX-1 and PGsyn was evaluated. Results showed that oral ketorolac treatment significantly reduced both enzymes; surprisingly, oral treatment with ketogal did not produce significant variation in the expression of the two constitutive enzymes. Moreover, histological experiments on stomach and kidneys clearly indicated that repeated administration of ketogal induced lower toxicity than ketorolac. At same time, *in vivo* results clearly showed that both ketorolac and ketogal had a similar therapeutic activity in a model of inflammation and in pain perception. These effects were accompanied by the reduction of enzyme expression such as COX-2 and iNOS, and by the modulation of levels of nuclear NF-κB and cytosolic IκB-α in the inflamed paws. These very encouraging results demonstrate for the first time that ketogal could represent a valid and novel therapeutic alternative to the ketorolac and might pave the way for clinical studies.

## Introduction

Non-steroidal anti-inflammatory drugs (NSAIDs) are the therapeutic agents of first choice for the treatment of inflammation, pain, and fever. The term NSAID refers to a group of structurally diverse chemical compounds that share the ability to inhibit the activity of the prostaglandin (PG) biosynthetic enzymes, the cyclooxygenase (COX) isoforms 1 and 2. It is shown that anti-inflammatory activity of NSAIDs is also due to degradation of IκB-α and nuclear translocation of NF-κB both *in vitro* and *in vivo* (Stark et al., 2001; Loveridge et al., 2008). Moreover, NF-κB stimulates the expression of enzymes whose products contribute to the pathogenesis of the inflammatory process, including the inducible form of nitric oxide synthase (iNOS) and the COX-2 (Pahl, 1999). Unfortunately, gastrointestinal (GI) toxicity still remains the biggest problem for current NSAIDs-based therapies. The number of new developed drugs approved annually continues to decline because of the problems related to pharmacological safety. It is the case of the selective COX-2 inhibitors that in the beginning were very promising because of their selective inhibitor effect, which reduced GI side effects. However, very soon the adverse cardiovascular effects have dramatically reduced their use in the clinical practice (Drazen, 2005; McGettigan and Henry, 2006). GI side effects produced by non-selective COX-1/-2 inhibitors are either due to direct contact or indirect effect of the drug on the gastrointestinal mucus membrane. Acidic nature of NSAIDs, ion trapping, and inhibition of cytoprotective prostaglandins are some of the reasons for the GI adverse effects (Cioli et al., 1979; Rainsford, 1989). Recently, a great attention has been paid to the derivatization of NSAIDs carboxyl group in order to develop gastro sparing prodrugs. Among the NSAIDs on the market, ketorolac appears a good candidate. This non-steroidal and non-narcotic drug is administered systemically (via oral and parenteral route) for the control of mild-to-moderate pain as well as of some postoperative and cancer pain, and its mechanisms are well known (O’Hara et al., 1987; Brown et al., 1990; Joishy and Walsh, 1998; Mercadante and Giarratano, 2013). Despite its high therapeutic potential, clinical use has been strongly limited because of the toxicity. In fact, long-term exposure to this drug has been correlated with an enhanced risk of gastrointestinal bleeding and renal failure (Litvak and McEvoy, 1990; Laporte et al., 2004; Boyer et al., 2010). For this reason, its safety profile has been carefully monitored during the last years and its use limited to the short-term treatments (Gillis and Brogden, 1997; Dula et al., 2000). Several evidences have shown that restricting the dosage and duration of exposure, as well as use in patients younger than 65 years old significantly reduces adverse effects, but this therapeutic approach is not always effective (Soleyman-Zomalan et al., 2017). Many efforts have been made to synthesize new prodrugs form ketorolac, by masking its carboxylic acid group (Suthar and Sharma, 2015), obtaining a minor gastrointestinal toxicity by contact. Moreover, to obtain a reduction in GI toxicity, it is important the role of endogenous enzymes, such as prostaglandin synthetase (PGsyn), and COX-1 (Miller and Jacobson, 1979; Brzozowski et al., 2005; Szabo, 2014). The observation that non-selective COX-1/-2 inhibitors block the activity of the PGsyn system both *in vitro* and *in vivo* suggests that GI toxicity encountered in humans treated with NSAIDs may be due to a reduction of COX-1 activity and deficiency of endogenous PGs (Miller and Jacobson, 1979). Moreover, the literature data also underline that altered expression of these two constitutive enzymes is necessary for maintenance of tissue homeostasis. In particular, recently, Mard et al. (2016) showed that the level of protein expression of COX-1 in indomethacin-treated animals was significantly lower than in normal untreated animals.

Based on these evidences, the aim of this paper was to evaluate gastric toxicity (by COX-1 and PGsyn expression and by histological study) and renal alteration (by metabolic cages and histological study) after repeated ketorolac and ketogal oral treatment in naïve mice. Moreover, therapeutic activity in acute (carrageenan) and chronic (incision paw) pain models was tested and pro-inflammatory enzyme expression (iNOS and COX-2) and mediators (NF-κB and IκB-α) were also evaluated.

## Materials and Methods

### Drugs and Chemicals

Ketorolac was purchased from commercial sources (Sigma–Aldrich, Milan, Italy) as free acid. Ketogal was synthesized as described (Magliocca et al., 2017). The synthesis was carried out with green strategies and involves two steps. First, esterification of ketorolac with 1,2,3,4-di-*O*-isopropylidene-D-α-galactopyranose (DIPG) in the presence of *N*-ethyl-*N*′-(3-dimethylaminopropyl)carbodiimide hydrochloride (EDC) as a condensing agent and 4-(dimethylamino) pyridine (DMAP), as catalyst in [bmim][PF_6_], then, ketals were completely removed in a mixture of ACN, hydrochloric acid 1N, and GAA at reflux for 40 min. Evaporation of the solvent gave a residue which was purified on a chromatography column with silica gel by using ethyl acetate as eluent so as to obtain ketogal. Indomethacin was obtained by Sigma–Aldrich, (Milan, Italy), and it was used only for histological experiments.

### Animals

The 10-week-old male Swiss CD1 mice (30–35 g) were purchased from Charles Rivers (Calco, Italy). They were housed in cages in a room kept at 22 ± 1°C on a 12 h:12 h light/dark cycle. All animals were acclimated to their environment for 1 week and had *ad libitum* access to water and standard rodent chow diet. All procedures involving mice were carried out in accordance with the Institutional Guidelines and complied with the Italian Ministry of Health and associated guidelines from European Communities Council Directive. The procedures reported here were approved by the Institutional Committee on the Ethics of Animal Experiments (CSV) of the University of Naples “Federico II” and by the Ministry of Health under protocol no. 2014-0084607. At the end of all experiments, the animals were euthanized by CO_2_ overdose.

## *In Vivo* Experiment

### Anti-inflammatory Activity

Paw edema was induced by a subplantar injection of λ–carrageenan (1%) into the right hind paw (D’Agostino et al., 2007). Vehicle, Ketorolac, and ketogal were orally administrated for 5 days. Paw volumes were measured by a plethysmometer apparatus (Ugo Basile, Italy) at different time intervals before injection (0) and 1, 2, 3, 6, and 24 h after administration of carrageenan. The increase of paw volume was evaluated as the difference between the paw volume measured at each time point and the basal paw volume measured immediately before carrageenan injection.

### Plantar Incision

Postoperative pain was induced by incision paw according to the method described by Pogatzki and Raja (2003). All mice were anesthetized with enflurane/O_2_ mixture and were maintained by a mask during the administration procedure. The left paw was disinfected with Betadine; a 0.5-cm longitudinal incision was made with a blade, through skin and fascia of the plantar aspect of the foot, starting 0.3 cm from the proximal edge of the heel and extending toward the toes. The underlying muscle was elevated with a curved forceps, leaving the muscle origin and insertion intact. The skin was closed with a single 8–0 nylon suture. The incisions were checked daily and any signs of wound infection or dehiscence excluded the animal from the study.

### Mechanical Hyperalgesia

Latencies of paw withdrawal (g) were evaluated by mechanical stimuli using the Randall–Selitto analgesiometer for mice (Ugo Basile, Italy, model 37216). Vehicle, Ketorolac, and Ketogal were orally administrated for 5 days; in carrageenan experiment, hyperalgesia was assessed on ipsilateral paw before (0) and 1, 2, 3, 6, and 24 h; while in postoperative study, mechanical pain was performed at day 5 following incision. Each paw was tested twice per session. Cutoff force was set at 100 g to avoid tissue injury to the animals.

### Thermal Hyperalgesia

Thermal hyperalgesia was examined by measuring the latency to withdrawal of the hind paws from a focused beam of radiant heat applied to the plantar surface using a Plantar Test apparatus (Ugo Basile, Milan, Italy). The days before experiment, animals were placed in a transparent Perspex box with a thin glass floor and allowed to acclimatize for 30 min. Ketorolac and ketogal were orally administrated for 5 days; withdrawal latencies to radiant heat were measured on inflamed paw at day 5 at different time intervals (1, 2, 3, 6, and 24 h). Cutoff was set at 30 s to avoid tissue injury to the animals.

### Volume of Urine

Fasting mice (12–14 h) were treated for 5 or 7 days with ketorolac (10 mg/kg/os), ketogal (16.6 mg/kg/os) or vehicle; 1 h following oral administration, mice were placed in metabolic cages for 24 h and the volume of urine was measured (*n* = 6). Results are expressed as the total amount of urine (mL) of 5 or 7 days.

## *Ex Vivo* Experiment

### Protein Extraction and Western Blot Analysis

To obtain cytosolic protein extracts, skin was removed and paws were homogenized in extraction buffer [0.32 M sucrose, 10 mM TRIS–HCl pH 7.4, 1 mM ethylene glycol-bis(β-aminoethyl ether)-N,N,N′,N′–tetraacetic acid (EGTA), 2 mM ethylenediaminetetraacetic acid (EDTA), 5 mM NaN_3_, 10 mM 2-mercaptoethanol, 50 mM NaF, 0.2 mM phenylmethylsulphonylfluoride (PMSF), 0,15 μM pepstatin A, 20 μM leupeptin, and 1 mM sodium orthovanadate]. The homogenates were chilled on ice for 15 min and then centrifuged at 1000 *g* for 10 min at 4°C; and the supernatant was stored at -80°C until use. To obtain cytosolic fraction, pellets were suspended in the supplied complete lysis buffer containing 1% Triton X-100, 150 mM NaCl, 10 mM Tris–HCl pH 7.4, 1 mM EGTA, 1 mM EDTA, 0,2 mM PMSF, 20 μM, 0,2 mM sodium orthovanadate and then centrifuged for 30 min at 15000 *g* at 4°C.

Frozen stomachs were rapidly homogenized in ice-cold hypotonic lysis buffer (10 mM HEPES, 1.5 mM MgCl_2_, 10 mM KCl, 0.5 mM PMSF, 1.5 μg/ml soybean trypsin inhibitor, 7 μg/ml pepstatin A, 5 μg/ml leupeptin, 0.1 mM benzamidine, 0.5 mM dithiothreitol) and incubated in ice for 45 min. The cytoplasmatic fraction was then obtained by centrifugation at 13,000 *g* for 15 min at 4°C.

Protein concentrations were estimated by the Bio-Rad protein assay using bovine serum albumin as standard. Paw (70 μg) and stomach (30 μg) lysate proteins were dissolved in Laemmli sample buffer, boiled for 5 min and separated on acrylamide gel electrophoresis and transferred to nitrocellulose membrane (240 mA for 40 min at room temperature). The filter was then blocked with 1X phosphate buffer saline (PBS) and 3% non-fat dried milk for 45 min at room temperature and probed with anti-cyclooxygenase (COX)-2 (dilution 1:1000; BD Bioscience, from Becton Dickinson, Buccinasco, Italy), anti-inducible nitric oxide synthase (iNOS) antibody (dilution 1:1000; BD Bioscience), anti-NF-κB p65 (dilution 1:500; Santa Cruz Biotechnology, Inc., Santa Cruz, CA, United States), or anti-IκB-α (dilution 1:500; Santa Cruz Biotechnology, Inc.) antibody for cytosolic and nuclear paw lysates. Stomach lysates were probed with anti-COX-1 (dilution 1:1000; Santa Cruz Biotechnology, Inc.) and anti-prostaglandin E (PGE)-synthase (dilution 1:1000; Santa Cruz Biotechnology, Inc.) antibody. The secondary antibody was incubated for 1 h at room temperature. The immune complex visualized by Image Quant (GE Healthcare, Milan, Italy). The protein bands were densitometrically analyzed with a model GS-700 imaging densitometer (Bio-Rad Laboratories, Milan, Italy). To ascertain that blots were loaded with equal amounts of protein lysates, they were also incubated in the presence of the antibody against the β-actin protein (clone AC-15; dilution 1:15000, Sigma–Aldrich).

### Ulcerogenicity Studies

Non-steroidal anti-inflammatory drugs (NSAIDs)-induced gastric damage in mice is evaluated following the procedure described by Chan et al., 1995. In fasted (16–18 h) mice (*n* = 6 for each group), ketorolac free acid (10 mg/kg), ketogal (16.3 mg/kg), or vehicle (CMC 0.5%) were administered orally for 5 days; at last day, mice were euthanized 1 h after treatment and the stomach was excised along its greater curvature, rinsed with normal saline, and the mucosa was examined by means of a magnifying glass for the presence of irritation or frank hemorrhagic lesions (ulcers). Irritation/ulcers were assigned a score from 0 to 3; 0 = no irritation, 3 = high irritation. The sum of total scores was used for comparison.

### Histological Evaluation

In another set of experiments, we evaluated histological aspect of stomach and kidneys from fasting mice (*n* = 6 for each group; 12–14 h) treated for 5 days with ketorolac (10 mg/kg/os), ketogal (16.3 mg/kg/os), indomethacin (15 mg/kg/os), and vehicle (CMC 0.5%), and from untreated fed mice. Mice were deeply anesthetized with pentobarbitone (50 mg/kg^-1^, IP) and transcardially perfused with PBS followed by 4% paraformaldehyde (PFA). Then, the stomachs and kidneys were excised and fixed in 10% buffered paraformaldehyde solution at 4°C for 24 h. Finally, the samples were processed for wax embedding according to routine protocols, serial sections were cut and stained with hematoxylin–eosin. Periodic acid–Schiff (PAS) was used to highlight mucin, glycogen, and glycoproteins. Sections were oxidized in 0.5% periodic acid solution for 10 min, rinsed in double-distilled water, and stained with Schiff’s reagent in the dark for 45 min. The reaction was blocked by repeated washing in 2.5% sodium bisulphite in 0.05 N HCl (Avallone et al., 2015).

### Statistical Analysis

Statistical analyses were performed on raw data using Prism 5 Graphpad software (GraphPad Software Inc., San Diego, CA, United States). *In vitro* data are presented as mean ± SEM, results was expressed as optical density (OD) (arbitrary units; mm^2^) and normalized against the expression of the housekeeping protein β-actin. The significance of differences between groups was determined by one-way repeated measurements ANOVA followed by *post hoc* Bonferroni’s *in vivo* data are presented as mean ± SEM; pain tests were expressed as paw withdrawal threshold (PWT) expressed in gram (g) for mechanical hyperalgesia and in second (s) for thermal hyperalgesia. The significance of differences between groups was determined by one- or two-way repeated measurements ANOVA followed by *post hoc* Bonferroni’s test. *P* < 0.05 was considered statistically significant for all tests.

## Results

### Evaluation of Gastric Toxicity after Repeated Oral Administration of Ketorolac or Ketogal

**FIGURE 1:**
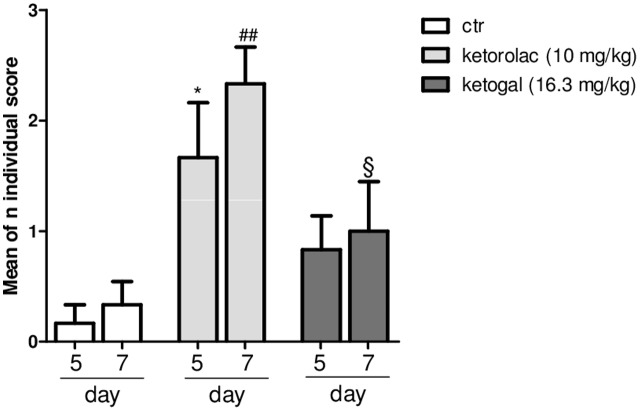
Evaluation of ulcerogenic activity of repeated (for 5 or 7 days) treatment of ketorolac (10 mg/kg/os, white–gray columns), ketogal (16.3 mg/kg/os, black–gray columns), and ctr (vehicle, os, white columns). Individual score from 0 to 3 was assigned; the sum of total scores was used for comparison. Data are shown as mean ± SEM of six animals per group. The significance of differences between groups was determined by one-way ANOVA followed by *post hoc* Bonferroni’s test. ^∗^*P* < 0.05 vs. ctr day 5; ^##^*P* < 0.01 vs. ctr day 7; ^x^*P* < 0.05 vs. ketorolac day 7.

**FIGURE 2:**
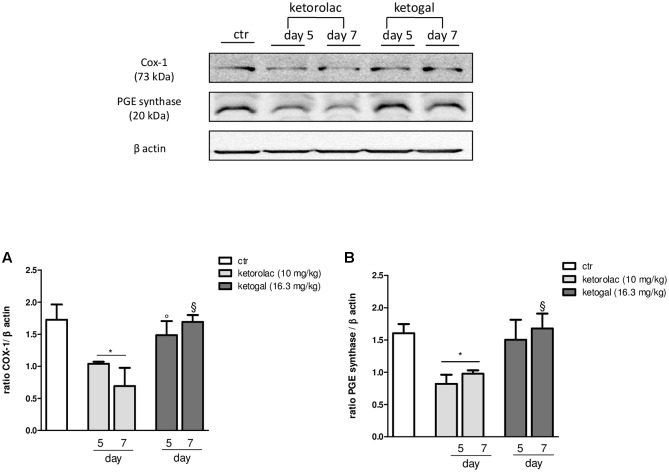
Modulation of COX-1 **(A)** and PGE synthase **(B)** expression in stomach of naïve mice at 5 and 7 days following oral ketorolac (10 mg/kg, white–gray columns), ketogal (16.3 mg/kg, black–gray columns), and ctr (vehicle, white columns) administration. Data are shown as mean ± SEM of 6 per group. The significance of differences between groups was determined by one-way ANOVA followed by *post hoc* Bonferroni’s test. ^∗^*P* < 0.05 vs. ctr day 5; °*P* < 0.05 vs. ketorolac day 5; ^x^*P* < 0.05 vs. ketorolac day 7.

**FIGURE 3:**
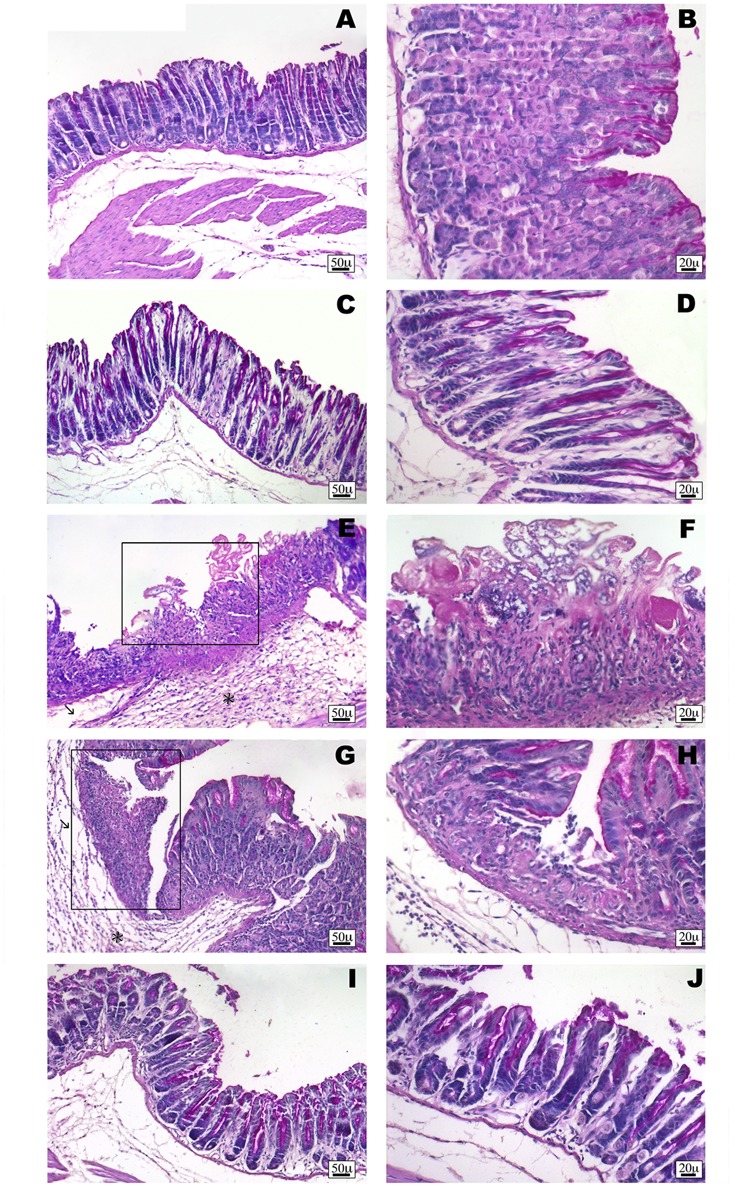
Histological observations of different treatments on the stomach of mouse, PAS staining. **(A,B)** White stomach, the gastric mucosa shows regular morphology with normal complement of mucosal lineage. **(C,D)** Vehicle-treated mice stomach presents normal histological appearance. Slight alterations are rarely observed. **(E,F)** Indomethacin-treated stomach has severe lesions of mucosa, with foveolar and parietal cells eroded (frame). Prominent inflammatory infiltrate (^∗^) and oedema (arrow) are evident. **(G,H)** Ketorolac-treated stomach shows necrotic area (frame) with a severe infiltrate (^∗^) and oedema (arrow). **(I,J)** Ketogal-treated stomach that shows the gastric mucosa has a slight alteration and normal mucus production.

Non-steroidal anti-inflammatory drug (NSAID) long-term administration induces significant stomach ulcers and histopathological changes in kidney, with consequent nephrotoxicity (Gooch et al., 2007). Our data confirm these hypothesis, since we showed that 5 or 7 days of oral ketorolac (10 mg/kg/os, gray–white columns) treatment provoked a considerable number of ulcers in stomach, compared to control group (gray–white columns) (**Figure 1**; ^∗^*P* < 0.05 vs. ctr day 5, and ^##^*P* < 0.01 vs. ctr day 7). By contrast, equimolecular oral dose of ketogal (16.6 mg/kg/os, gray–black columns) did not induce an increase of numbers of ulcers compared to vehicle-treated mice, but it is noteworthy lower if compared to ketorolac (**Figure 1**). Moreover, ketogal-treated mice showed following 7 treatment days, a lower ulcers formation if compared to ketorolac (**Figure 1**; ^x^*P* < 0.05 vs. ketorolac day 7). To confirm of these data, we evaluated COX-1 and PGsyn expression in the stomach of naïve mice trough WB analysis (**Figure 2**); results showed that COX-1 (**Figure 2A**) and PGEsyn (**Figure 2B**) expression was significantly reduced in ketorolac-treated mice following 5 and 7 days (**Figures 2A,B**, gray–white columns; ^∗^*P* < 0.05 vs. ctr), while oral equimolecular dose of ketogal surprisingly did not produce significant variation of these two constitutive enzymes. Significant difference was observed between ketorolac group and ketogal-treated mice, to confirm that our prodrug has a better toxicological profile (**Figure 2**; °*P* < 0.05 vs. ketorolac day 5 and ^x^*P* < 0.05 vs. ketorolac day 7). These data were also supported by histological analysis. Light microscopy observations on the stomach, after PAS staining, showed that in untreated mice the gastric mucosa had a normal complement of mucosal lineage: chief cells that produce pepsinogen, parietal cells in the intermediate zone, hydrogen chloride secreting, and foveolar mucus-producing cells (**Figures 3A,B**). Also vehicle-treated mice had a regular morphology with normal mucus production and a lack of inflammatory infiltrate; slight alterations are rarely observed (**Figures 3C,D**). The stomach treated with indomethacin (15 mg/kg/os), used as positive control, exhibits severe lesions of mucosa, mainly at the level of foveolar and parietal cells that are eroded (ulcers) with an inflammatory infiltrate in the lower part of mucosa and sub-mucosa (**Figures 3E,F**). Stomach treated with ketorolac (10 mg/kg/os for 5 days) presented necrotic areas with the complete loss of the three cell types and an evident inflammatory infiltrate; ulcer areas with an initial disintegration of the mucosal surface, particularly at the level of cells foveolar, were evident as well (**Figures 3G,H**). Finally, gastric mucosa of mice treated with ketogal (16.3 mg/kg/os for 5 days) showed a considerable improvement in the response to the drug with slight alterations and normal mucus production (**Figures 3I,J**).

### Evaluation of Kidney Toxicity after Repeated Oral Administration of Ketorolac or Ketogal

**FIGURE 4:**
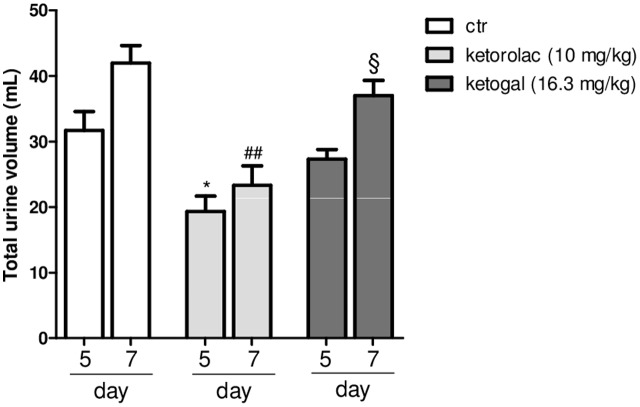
Effect of 5 or 7 days of oral ketorolac (10 mg/kg, white–gray columns), ketogal (16.3 mg/kg, black–gray columns), and ctr (vehicle, white columns) administration on total urine volume in naïve mice. Data are shown as mean ± SEM of six animals per group. The significance of differences between groups was determined by one-way ANOVA followed by *post hoc* Bonferroni’s test. ^∗^*P* < 0.05 vs. ctr day 5; ^##^*P* < 0.01 vs. ctr day 7; ^x^*P* < 0.05 vs. ketorolac day 7.

**FIGURE 5:**
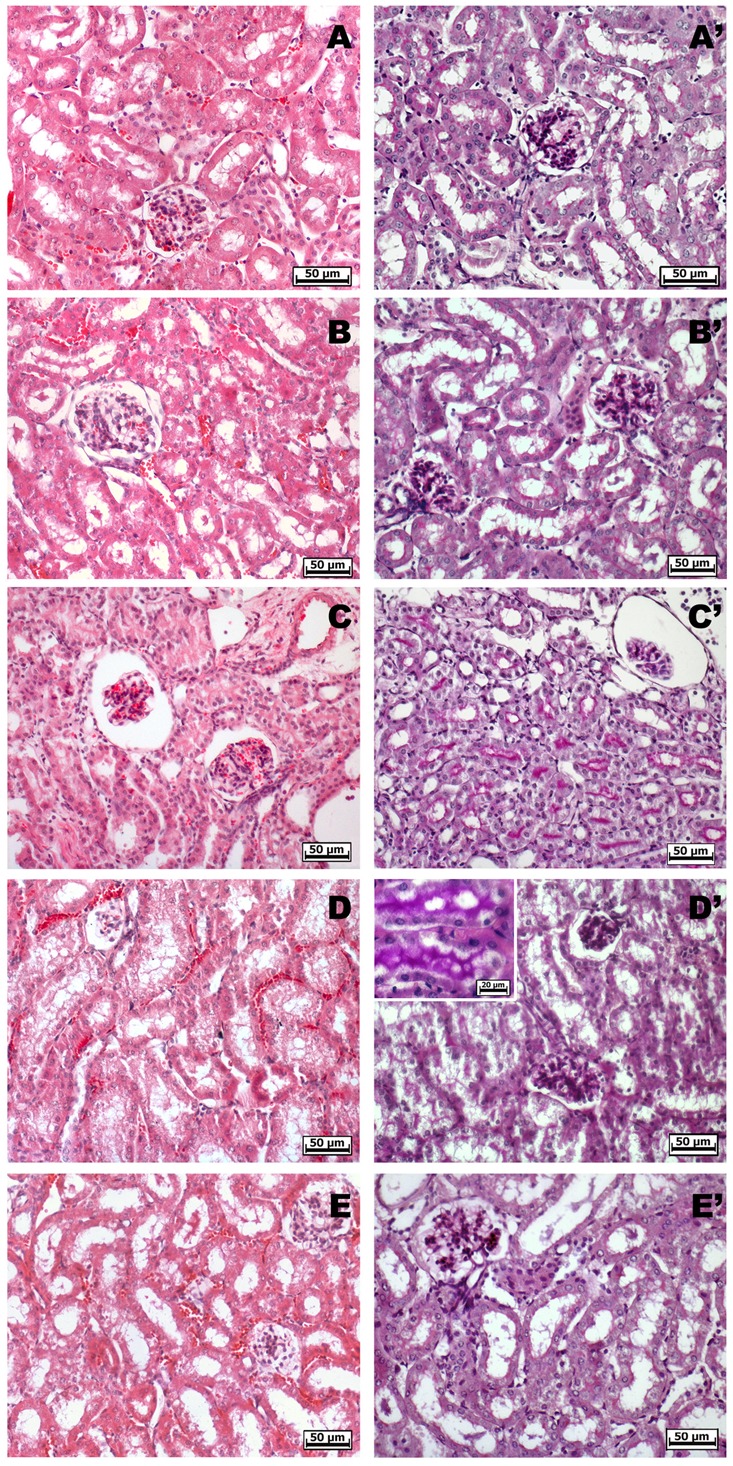
Histological observations of different treatments on the kidney of mouse, hematoxylin-eosin **(A–E)** – PAS staining **(A′–E′)**. **(A,A′)** White and **(B,B′)** vehicle-treated mice kidney, they show the typical organization in nephrons. **(C,C′)** Indomethacin-treated kidney highlights contracted glomeruli, a slight cytoplasmic vacuolization of the tubular epithelium and a higher PAS positivity. **(D,D′)** Ketorolac-treated kidney shows many contracted glomeruli, marked cytoplasmic vacuolization and a strong PAS positivity of the tubular epithelium. **(E,E′)** Ketogal-treated kidney exhibits a normal morphology of the nephron such as the positivity of the neutral mucopolysaccharides.

Non-steroidal anti-inflammatory drugs (NSAIDs) alter renal function through their effects on renal PGs, and these become critically involved in the control of renal hemodynamics and in maintaining the glomerular filtration rate (GFR) (Radi, 2012). We evaluated the total volume of urine in mice treated with ketorolac (10 mg/kg/os) or ketogal (16.3 mg/kg/os) and we found that oral repeated ketorolac treatment significantly reduced the total urine volume at 5 and 7 days (**Figure 4**, gray–white columns, ^∗^*P* < 0.05 vs. ctr day 5 and ^##^*P* < 0.01 vs. ctr day 7), while oral ketogal treatment did not produce any significant effect on urine volume compared to control group (**Figure 4**, black–white columns). Ketogal-treated mice showed following 7 treatment days, a more high volume of urine if compared to ketorolac (**Figure 4**; ^x^*P* < 0.05 vs. ketorolac day 7), data that underline a best safety profile respect its parent drug. To confirm these data, histological studies were performed. Hematoxylin-eosin and PAS staining were used to highlight different kind of damage (**Figure 5**). Our study focused on the glomerular morphology and the evaluation of neutral mucopolysaccharides. Light microscopy observations showed that in the untreated and vehicle-treated mice, the kidney maintains the typical organization in nephrons, each one composed of the glomerulus, proximal tubule, descending and ascending loops of Henle, straight segment, macula densa, and distal tubule (**Figures 5A–B**′). Kidney treated with indomethacin (15 mg/kg/os) display contracted glomeruli detached from Bowman’s capsule, and a slight cytoplasmic vacuolization of the tubular epithelium (**Figures 5C,C**′). PAS positivity is more marked in this treatment and the mucopolysaccharides seem to fill completely the tubular lumen which appears already reduced (**Figure 5C**′). Kidney treated with ketorolac (10 mg/kg/os for 5 days) showed a loss of consistency and a more marked cytoplasmic vacuolization of the tubular epithelium. Many glomeruli are contracted and there is an increase in PAS positivity of some tubules (**Figures 5D,D**′). Lastly kidney treated with ketogal exhibits a remarkable restoration of the normal morphology of the nephron (**Figures 5E,E**′), even though some contracted corpuscles are still present. The neutral mucopolysaccharides appear normal (**Figure 5E**′).

### Anti-inflammatory and Anti-hyperalgesic Effects of Repeated Oral Ketorolac or Ketogal Administration

**FIGURE 6:**
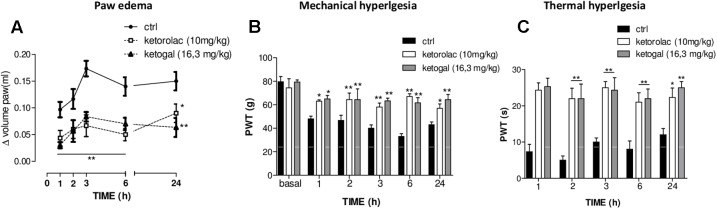
Effect of 5 days of oral ketorolac (10 mg/kg), ketogal (16.3 mg/kg), and ctr (vehicle) administration in paw oedema **(A)**, mechanical **(B),** and thermal **(C)** hyperalgesia by carrageenan. Pharmacological activity was evaluated at 1–2–3–6–24 h following drugs administration; basal represent values before carrageenan injection. Data are shown as mean ± SEM of six animals per group. The significance of differences between groups was determined by two-way ANOVA followed by *post hoc* Bonferroni’s test. ^∗^*P* < 0.05, ^∗∗^*P* < 0.01 vs. ctr.

**FIGURE 7:**
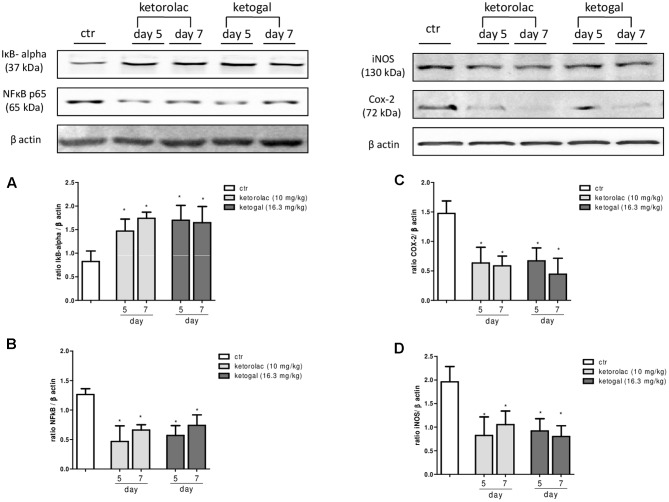
Evaluation of IkB-α degradation **(A)**, the translocation of the p65 subunit of NF-kB into the nucleus **(B)**, COX-2 **(C)**, and iNOS **(D)** enzymes expression in the carrageenan-induced paw oedema at 5 and 7 days following oral ketorolac (10 mg/kg, white–gray columns), ketogal (16.3 mg/kg, black–gray columns), and ctr (vehicle, white columns) administration. Data are shown as mean ± SEM of six animals per group. The significance of differences between groups was determined by one-way ANOVA followed by *post hoc* Bonferroni’s test. ^∗^*P* < 0.05 vs. ctr.

**FIGURE 8:**
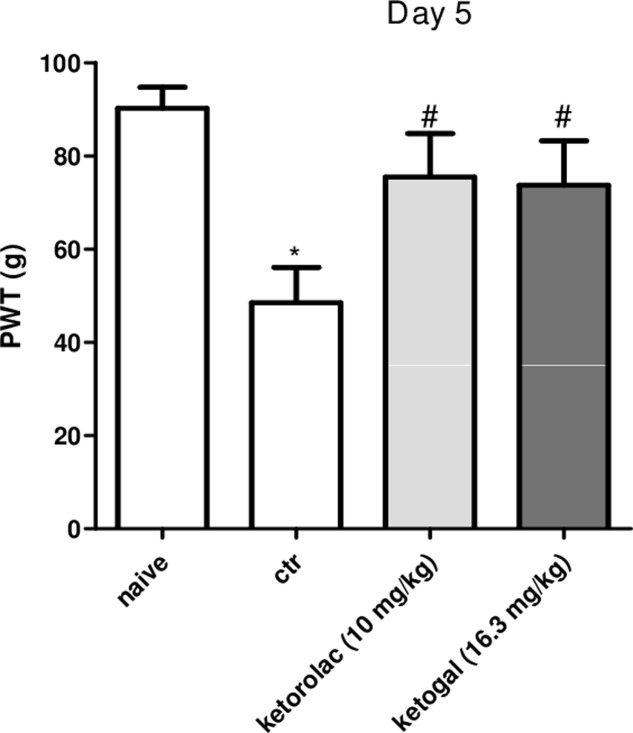
Effect of 5 days of oral ketorolac (10 mg/kg, white–gray columns), ketogal (16.3 mg/kg, black–gray columns), and ctr (vehicle, white columns) administration in postoperative pain by paw’s incision. Naïve represent mice without surgery. Data are shown as mean ± SEM of six animals per group. The significance of differences between groups was determined by one-way ANOVA followed by *post hoc* Bonferroni’s test. ^∗^*P* < 0.05 vs. naïve; ^#^*P* < 0.0 vs. ctr.

We also evaluated therapeutic properties of two drugs following repeated administration. Intraplantar injection of carrageenan induced both paw edema and hyperalgesia (**Figure 6A**, black circle; **Figures 6B,C**, black column), as expected, repeated oral administration for 5 days with ketorolac (10 mg/kg) reduced paw edema (**Figure 6A**) and mechanical (**Figure 6B**) and thermal (**Figure 6C**) hyperalgesia at all experimental points (1, 2, 3, 6, and 24 h). Equimolecular doses of ketogal (16.3 mg/kg) showed similar anti-inflammatory and analgesic activities. Similar results were obtained also after 7 days of treatment (data not shown). In order to investigate the mechanism by which ketorolac and ketogal attenuate the inflammatory pathway involved in both edema and pain perception, we analyzed cytosolic COX-2 and iNOS expression and IκB-α and nuclear NF-κB p65 extracts of inflamed paws. On days 5 and 7, ketorolac and ketogal significantly prevented IκB-α degradation (**Figure 7A**) and the translocation of the p65 subunit of NF-κB into the nucleus (**Figure 7B**). Inflammatory enzymes and cytokines, whose expression depends on the transcriptional NF-κB activation, were also determined. As shown in **Figures 7C,D**, ketorolac and ketogal, respectively, reduced significantly COX-2 and iNOS expression in the inflamed paws. Finally, we tested two drugs in postoperative pain; following incision of paw, operated vehicle–animals showed signs of hyperalgesia if compared to naïve mice (**Figure 8**; ^∗^*P* < 0.05 vs. naïve). At day 5, after repeated oral treatment, ketorolac (10 mg/kg/os) and ketogal (16.3 mg/kg/os) showed a significant anti-hyperalgesic effect (**Figure 8**; ^#^*P* < 0.05 vs. ctr). No effects were observed on the contralateral paws (non-operated paw) (data not shown).

## Discussion

In this paper, we clearly show that repeated administration of ketorolac–galactose conjugate (ketogal) exerted a significant therapeutic activity, similar to its parent drug ketorolac, but with lower toxicity. In particular, our data demonstrated that using an experimental protocol characterized by repeated ketorolac administration for at least 5 days, treatment that is usually used in cause of chronic inflammation or in cause of postoperative pain, a noteworthy gastric and renal toxicity was induced. Surprisingly, in same experimental conditions, ketogal showed a remarkable safety profile while maintaining therapeutic activities of its parent drug.

Gastrointestinal (GI) toxicity exerted by NSAIDs owes more mechanisms of action and among them there is the direct contact of carboxylic group with gastric mucosa, a phenomenon called ion-trapping. Masking the carboxylic group with a vector reduces the contact with mucosa and this finally decreases at least in part the toxicity of the NSAID (Sigthorsson et al., 2000). In our case, the esterification with galactose, not only improves pharmacokinetic profile of ketorolac, but also increases its therapeutic activity (Curcio et al., 2009). To the best of our knowledge, none of the ketorolac’s prodrug reported in previous studies has ever shown this important and unexpected effect. In a recent review, it has been raised criticism on the approaches and strategies employed in the last two decades to design and develop safer NSAIDs, which mainly focused on the amelioration of GI toxicity (Suthar and Sharma, 2015). Another important mechanism through which NSAIDs induce toxicity, after absorption and metabolism, is due to alteration the activities of PGs (Gambaro and Perazella, 2003; Huerta et al., 2005). Although several factors have been postulated as pathogenic elements of NSAID-induced GI damage, a deficiency of PGs has clearly been shown to play a critical role in the pathogenesis of these lesions (Whittle, 1981; Tanaka et al., 2002; Park et al., 2006). In fact, the correlation between NSAID and GI toxicity has been correlated with their ability to inhibit mucosal PG synthesis (Tanaka et al., 2001). It as well known that a long-term use of non-selective COX-1/-2 inhibitors results in gastric ulcers due to loss of physiological COX activity and to reduction of PGs production (Miller and Jacobson, 1979; Vane and Botting, 1995; Brzozowski et al., 2005; Szabo, 2014). Novel findings suggest that for maintenance of physiological functions and tissue homeostasis not only it is important for the evaluation of COX-1 and PGsyn activity, but also a significant role is also due to evaluation of protein expression of these constitutive enzymes (Mard et al., 2016). Here, for the first time we showed that ketorolac-treated animals produced a significant reduction of COX-1 and PGsyn expressions in naïve mice if compared to vehicle group, probably through genetic mechanism, as others NSAIDs reported in the literature data (Shinji et al., 2005; Korotkova and Jakobsson, 2011; Calvello et al., 2012; Mard et al., 2016). It is well known that products of the COX pathway, such as PGs, are ligands of peroxisome proliferator-activated receptors (PPARs) (Kliewer et al., 1994; Forman et al., 1995; Scher and Pillinger, 2009). Probably, a minor activity of PGs induces an alteration in PPARs pathways. Several manuscripts showed the potential activity of these nuclear receptors in numerous pathologies, underline the necessity to keep the activity of these receptors to preserve the physiological integrity and wellness (Keller et al., 2000; Ehrmann et al., 2002). Surprisingly, repeated treatment of ketogal did not produce significant variation of expression of these two constitutive enzymes respect to vehicle group. These data were confirmed by histological experiments on stomach; in fact, ketogal-treated mice showed stomach tissue less damaged, ulcers significantly reduced, and normal mucus production, whereas in ketorolac-treated mice, necrotic and ulcer areas with an initial disintegration of the mucosal surface were evident. Finally, based on these last evidences and on lessened “topical” effect, thank to esterification of the carboxylic group with galactose, our derivate is effectively safer than its parent drug.

Miller and Jacobson (1979) were the first to underline the important role of PG synthetase in the integrity of stomach, the observation that non-selective COX-1/-2 inhibitors block the activity of this enzyme suggest that GI toxicity by NSAIDs may be due to a deficiency of endogenous PG. In light of this effect, the current pharmacological therapy involves the use of supplementation with PGE_2_ derivative to prevent COX-1/-2 inhibitor-induced GI toxicity (Kunikata et al., 2001), or use COX-2 selective inhibitors. About this approach with selective COX-2 inhibitors, it is well known that these drugs raise important questions about the cardiovascular toxicity. In addition, the inhibition of both COX-1 and COX-2 was required for NSAID-induced GI toxicity injury, suggesting a role of COX-2 as well as COX-1 in maintaining the mucosal integrity of these tissues (Tanaka et al., 2002; Laine et al., 2008). Therefore, the advantage of using a non-selective COX inhibitors with low toxicity still remains a goal of many preclinical researches. As previously reported, ketogal is not a simple prodrug, but it could be looked at as a new chemical entity (Melisi et al., 2011; Cardani et al., 2014). Several manuscripts on NSAIDs prodrugs obtained blocking the free carboxylic group of NSAIDs only argue the gastrolesivity and neglect others crucial side effects (Szabo, 2014; Suthar and Sharma, 2015). To the difference of these papers, in our study we also evaluated renal toxicity. As aspect, 5 or 7 days of ketorolac administration induced a significant kidney toxicity, evaluated by reduction of urine volume and by histological study. Surprisingly, ketogal administration did not produce any significant effect on urine volume, and a better morphology of the nephron was observed, confirming further that our prodrug has a better safety profile than its parent drug. The other aim of this study was to evaluate therapeutic efficacy of ketogal following repeated treatment. Ketorolac is today used for inflammation and for the control of mild to moderate pain as well as of some postoperative (O’Hara et al., 1987; Brown et al., 1990; Joishy and Walsh, 1998; Mercadante and Giarratano, 2013). Here we reproduced animal models of these two commune pathologies by carrageenan-induced inflammation and by incision paw-induced postoperative pain. Our data clearly showed that prodrug was able to reduce the inflammatory process and pain perception, as well as ketorolac. In particular, our results showed that ketorolac and equimolecular dose of ketogal reduced both inflammation and pain induced by carrageenan, and that repeated treatment was also effective in postoperative pain. These effects was confirmed by the reduction of pro-inflammatory enzymes such as COX-2 and iNOS, and by the modulation of expression of nuclear NF-κB and cytosolic IκB-α. It is well known that these factors play a key role in inflammatory process both *in vitro* (Jang et al., 2017) and *in vivo* (Philkhana et al., 2017), and their reduction supports our hypothesis that ketogal, such as ketorolac, has a significant anti-inflammatory effect. These very encouraging results demonstrate for the first time that ketogal could be a valid and novel therapeutic alternative to the ketorolac. In addition, a prodrug of a powerful NSAID, as ketorolac, which preserves its pharmacological activity, can be of great interest to decrease the number of hospitalizations and to avoid the use of gastroprotective drugs, such as proton pump inhibitors, themselves not devoid of toxicity (Targownik et al., 2008; Wallace et al., 2011). In this scenario, the exploitation of a well-known compound already on the market and coupled with a non-toxic molecule, i.e., galactose, is undoubtedly an effective solution in terms of economy, safety, and efficacy for patients.

## Conclusion

We demonstrated that ketogal is a valid substitute of its original molecule ketorolac, due to the balance between therapeutic and toxic effects. Though further investigations are necessary to better characterize ketogal features, we strongly believe that it might be successfully tested in clinical studies.

## Author Contributions

RR, AC, MR, GB, and RC participated in research design. RR, CDC, SM, MN, RT, CaC, BA, CA, ClC, GS, and GR conducted experiments. SM, MR, MN, and GB contributed new reagents or analytic tools. RR, CaC, and BA performed data analysis. RR, MR, and BA wrote or contributed to the writing of the manuscript.

## Conflict of Interest Statement

The authors declare that the research was conducted in the absence of any commercial or financial relationships that could be construed as a potential conflict of interest.

## Abbreviations

ACN: acetonitrile
[bmim][PF_6_]: 1-butyl-3-methylimidazolium hexafluorophosphate
CMC: carboxymethyl cellulose
COX: cyclooxygenase
DIPG: 1,2,3,4-di-*O*-isopropylidene-D-α-galactopyranose
DMAP: 4-(dimethylamino)pyridine
EDC: *N*-ethyl-*N*′-(3-dimethylaminopropyl) carbodiimide hydrochloride
GAA: glacial acetic acid
GFR: glomerular filtration rate
GI: gastrointestinal
i.p.: intraperitoneal
MRT: mean residence time
NSAIDs: non-steroidal anti-inflammatory drugs
PAS: periodic acid–Schiff
PBS: phosphate buffered saline PFA paraformaldehyde
PGs: prostaglandins
PGsyn: prostaglandin synthetase
PT: Plantar Test
PWT: Paw withdrawal threshold
RPM: revolutions per minute
TFA: trifluoroacetic acid.
